# Anti‐inflammatory effect of *Trichospira verticillata* via suppression of the NLRP3 inflammasome in neutrophilic asthma

**DOI:** 10.1111/jcmm.18356

**Published:** 2024-04-26

**Authors:** Hyeyun Yang, Gunwoo Park, Sojung Lee, Sumin Lee, YeJi Kim, Nelson A. Zamora, Dong‐Keun Yi, Soo‐Yong Kim, Chun Whan Choi, Sangho Choi, Yong Hwan Park

**Affiliations:** ^1^ Department of Microbiology Ajou University School of Medicine Suwon Republic of Korea; ^2^ Department of Biomedical Sciences Graduate School of Ajou University Suwon Republic of Korea; ^3^ Department of Allergy and Clinical Immunology Ajou University School of Medicine Suwon Republic of Korea; ^4^ Instituto Nacional de Biodiversidad (INBio) Santo Domingo Costa Rica; ^5^ International Biological Material Research Center Korea Research Institute of Bioscience and Biotechnology Daejeon Republic of Korea; ^6^ Natural Biomaterial Team Gyeonggi Bio‐Center Suwon Republic of Korea

**Keywords:** inflammation, interleukin‐1β, neutrophilic asthma, NLRP3 inflammasome, *Trichospira verticillata*

## Abstract

*Trichospira verticillata* is an annual herb that belongs to the family *Asteraceae*. *Trichospira verticillata* extract (TVE) elicits anti‐plasmodial activity; however, there has been no detailed report about its anti‐inflammatory effects and molecular mechanisms. In addition, herbal plants exhibit anti‐inflammatory effects by suppressing the NLRP3 inflammasome. Therefore, the primary goal of this study was to examine the effects of TVE on NLRP3 inflammasome activation by measuring interleukin‐1β (IL‐1β) secretion. We treated lipopolysaccharides (LPS)‐primed J774A.1 and THP‐1 cells with TVE, which attenuated NLRP3 inflammasome activation. Notably, TVE did not affect nuclear factor‐kappa B (NF‐κB) signalling or intracellular reactive oxygen species (ROS) production and potassium efflux, suggesting that it inactivates the NLRP3 inflammasome via other mechanisms. Moreover, TVE suppressed the formation of apoptosis‐associated speck‐like protein (ASC) speck and oligomerization. Immunoprecipitation data revealed that TVE reduced the binding of NLRP3 to NIMA‐related kinase 7 (NEK7), resulting in reduced ASC oligomerization and speck formation. Moreover, TVE alleviated neutrophilic asthma (NA) symptoms in mice. This study demonstrates that TVE modulates the binding of NLPR3 to NEK7, thereby reporting novel insights into the mechanism by which TVE inhibits NLRP3 inflammasome. These findings suggest TVE as a potential therapeutic of NLRP3 inflammasome‐mediated diseases, particularly NA.

## 1 INTRODUCTION

*Trichospira verticillata* (L.) S.F. Blake is an herbaceous annual plant of the *Trichospira* genus, which is classified as a monotypic genus in the *Asteraceae* family. It is distributed in Tropical America and thrives mainly in seasonally dry tropical regions. Members of the *Asteraceae* family contain a wide range of medicinally active compounds, including anti‐inflammatory compounds. Dichloromethane extract of *T. verticillata* exhibits effective antiplasmodial activity^1^; however, its anti‐inflammatory function has not been investigated.

The cytokine interleukin‐1β (IL‐1β) is associated with both acute and chronic inflammation and plays a crucial role in the pathogenesis of human diseases. Consequently, the regulation of IL‐1β has drawn considerable attention. Cleaved and active IL‐1β are produced by the inflammasome, which is a caspase‐1‐activating multiprotein platform that responds to various stimuli.^2^ Among the inflammasomes, the NOD‐, LRR‐, and pyrin domain‐containing protein 3 (NLRP3) inflammasome has been intensively investigated due to its possible involvement in several human diseases. The NLRP3 is one of the cytosolic pattern recognition receptors (PRRs) that sense various pathogens or damage‐associated molecular patterns (PAMPs or DAMPs), such as pore‐forming toxins and uric acid. Activated NLRP3 recruits the adapter apoptosis‐associated speck‐like protein (ASC) protein, resulting in the proteolytic self‐cleavage and activation of pro‐caspase‐1. Active caspase‐1 induces the secretion of IL‐1β, resulting in pyroptosis. NLRP3 activation is tightly regulated by a two‐step process involving both priming and activating signals.^3^, ^4^ Mutations in the NLRP3 gene^5^, ^6^ cause autoinflammatory diseases called cryopyrin‐associated periodic syndromes (CAPSs).^7^, ^8^ Furthermore, the dysregulated activation of the NLRP3 inflammasome has been associated with the development of inflammatory diseases, including asthma,^9^ diabetes,^10^ atherosclerosis,^11^ and Alzheimer's disease.^12^, ^13^ As NLRP3 activity contributes to the pathogenesis of both rare autoinflammatory and common diseases, therapeutic targeting of the NLRP3 holds great promise for the treatment of many diseases. Although many studies have reported that small molecules inhibit the NLRP3 inflammasome by binding to the NLRP3 protein directly and that these inhibitors can alleviate inflammation in a disease mouse model,^14^ there is currently no approved drug to suppress the NLRP3 inflammasome.

In recent years, herbal plants have been found to exhibit anti‐inflammatory effects through the modulation of the NLRP3 inflammasome. Owing to their potent pharmacological activities, low toxicity, and cost‐effectiveness, they hold potential as valuable resources for the development of new drugs.

Neutrophilic asthma (NA) is a severe form of asthma with no known cure, and abnormal activation of the NLRP3 inflammasome is implicated in the development of NA.^14^ Therefore, it is crucial to screen for bioactive natural inhibitors of plant extracts and identify those that modulate NLRP3 activity to reduce inflammation, which could potentially become a novel therapeutic approach for NA. The present study aimed to investigate the anti‐inflammatory function of *T. verticillata* extract by conducting an inflammasome inhibition assay and to explore its potential application as a treatment for NA.

## 2 MATERIALS AND METHODS

### 2.1 Reagents and antibodies

*Trichospira verticillata* (L.) S.F. Blake Extract (TVE) was provided by Dong‐Keun Yi (International Biological Material Research Center, IBMRC). Penicillin–streptomycin, Foetal bovine serum (FBS), Dulbecco's Modified Eagle medium (DMEM), Roswell Park Memorial Institute (RPMI) 1640, pure down protein A/G‐agarose (P9203‐200), and phosphatase inhibitor cocktail (P3200‐005) were purchased from GenDEPOT (Baker, TX, USA). Antibodies against ASC (sc‐514414), c‐Myc (9E10) (sc‐40), NIMA‐related kinase 7 (NEK7; B‐5) (sc‐393539), and alpha‐tubulin (sc‐5286) were purchased from Santa Cruz Biotechnology (Santa Cruz, CA, USA). Mouse IL‐1β (AF‐401‐NA) and human IL‐1β antibodies (AF‐201‐NA) were purchased from R&D Systems (Minneapolis, MN, USA). Caspase‐1 (A0964) was purchased from ABclonal Technology (Woburn, MA, USA). Phospho‐NF‐κB p65 (93H1), NF‐κB p65 (8242), p‐ERK (9212S), and p44/42 MAPK (ERK1/2) (9101S) were purchased from Cell Signalling Technology (Danvers, MA, USA). Lipopolysaccharide from *P. gingivalis* (LPS, Ultrapure) (tlrl‐ppglps), ATP (tlrl‐atpl), nigericin (tlrl‐nig), monosodium urate crystals (MSU; tlrl‐msu), and flagellin (FLA‐ST; tlrl‐epstfla) were purchased from InvivoGen (San Diego, CA, USA). Lipofectamine 3000™ (L3000015) and highly cross‐adsorbed Mouse IgG (H + L) secondary antibodies (A32723) were purchased from Invitrogen (Thermo Fisher Scientific, Waltham, MA, USA). Imiquimod (IMQ; HY‐B0180) and phorbol 12‐myristate 13‐acetate (PMA; HY‐18739) were purchased from MedChem Express (South Brunswick Township, NJ, USA). EZ‐CYTOX (EZ‐500) was purchased from DoGenBio (Shanghai, China). Disuccinimidyl suberate (DSS; 68,528–80‐3) and N‐acetyl‐l‐cysteine (NAC; A9165‐25G) were purchased from Sigma‐Aldrich (St Louis., MO, USA). The Cellular Reactive Oxygen Species (ROS) Assay Kit (DCFDA/H2DCFDA) and nuclei stained with mounting medium (DAPI) (ab104139) were purchased from Abcam (ab113851; Cambridge, UK). The protease inhibitor cocktail was purchased from Quartett GmbH (PPI1015; Berlin, Germany). NE‐PER™ Nuclear and Cytoplasmic Extraction Reagents (78833) were purchased from Thermo Fisher Scientific. ADP‐Glo Kinase Assay (V6930), pGL4.32 Luciferase Reporter Vector (E8491), and Bright‐Glo™ Luciferase Assay System (E2610) were purchased from Promega (Madison, WI, USA). Mouse IL‐1beta/IL‐1F2 DuoSet Enzyme‐linked immunosorbent assay (ELISA; DY401), Mouse TNF‐alpha DuoSet ELISA (DY410), Mouse IL‐6 DuoSet ELISA (DY406), and Human IL‐1beta/IL‐1F2 DuoSet ELISA (DY201) were purchased from R&D systems.

### 2.2 Cell culture and stimulation

The immune cell lines, THP‐1 and J774A.1, were collected and cultured as previously described (ATCC). THP‐1 cells were differentiated by PMA (500 nM) for 3 h and then incubated for 2 days. J774A.1 cells were incubated for 1 day. For co‐culture experiments, A549 cells were plated into a transwell plate 1 day prior; then PMA‐differentiated THP‐1 cells were added to the A549 cells at 1:10 ratio. Subsequently, the cells were primed with LPS (100 ng/mL) for 5 h. Three hours after the cells were treated with LPS, TVE (10 μg/mL, 50 μg/mL, and 100 μg/mL) was further treated for 2 h. The cells were then stimulated with ATP (5 mM) for 30 min or 1 h, nigericin (10 μM) and MCC950 (100 nM) for 1 h, Imiquimod (200 μM) for 1 h, and monosodium urate crystals (100 μg/mL) for 3 h or transfected for 2 h with dsDNA (2 μg/mL) or for 3 h with flagellin (1.25 μg/mL) using Lipofectamine 3000™.

### 2.3 In vitro cell viability assay

Differentiated THP‐1 cells were seeded 2 days before the experiment and J774A.1 cells were seeded 1 day before the experiment in a 96‐well cell culture plate. The cells were primed with LPS for 5 h with or without TVE (10 μg/mL, 50 μg/mL, and 100 μg/mL) for 2 h, and then incubated with EZ‐CYTOX for 30 min. A549 cells were seeded in a 96‐well culture plate 1 day prior to the experiment. The cells were treated with or without TVE (10 μg/mL, 50 μg/mL, and 100 μg/mL) for 2 h or overnight and then incubated with EZ‐CYTOX for 1 h. The absorbance of the 96‐well plates was measured at 450 nm using an iMark™ Microplate Absorbance Reader (1681130; Bio‐Rad Laboratories, Hercules, CA, USA).

### 2.4 ASC oligomerization and ASC speck staining

For ASC oligomerization, pellets of THP‐1 cells were washed with phosphate‐buffered saline (PBS) and cross‐linked with DSS (2.5 mM). The mixture was incubated for 30 min at 25°C. The pellets were analysed by immunoblotting.

For ASC speck staining, NLRP3 inflammasome‐activated J774A.1 cells were fixed with 4% paraformaldehyde in PBS for 10 min at 25°C and washed three times with ice‐cold PBS. The cells were permeabilized with PBS containing 0.1% Triton X‐100 for 10 min at 25°C and washed three times with ice‐cold PBS for 5 min. Subsequently, cells were blocked with 1% bovine serum albumin (BSA) and 22.52 mg/mL glycine in PBS‐T for 30 min. Cells were incubated with ASC antibody overnight at 4°C. The cells were then washed three times with PBS for 5 min each. They were incubated with AF488‐conjugated anti‐mouse IgG antibody in 1% BSA for 1 h at 25°C in the dark. Then, they were washed three times with PBS for 5 min each. Nuclei were stained with a mounting medium (with DAPI). All images were captured using a confocal laser‐scanning microscope (LSM710; Carl Zeiss, Jena, Germany).

### 2.5 Extraction of nuclear proteins and cytosolic proteins

LPS‐primed J774A.1 cells (8 × 10^6^ cells) were treated with or without TVE (50 μg/mL and 100 μg/mL) for 2 h. Cells were collected in microtubes, and cell pellets were washed with PBS. The pellets were centrifuged at 500 × *g* for 3 min, and the supernatant was discarded. Cell pellets were homogenised in ice‐cold CERI, and protease/phosphatase inhibitor cocktails were added. The microtubes were vortexed vigorously at the highest setting for 20 s and incubated on ice for 10 min. Subsequently, the chilled CERII was added to the tubes, vortexed for 5 s, and incubated on ice for 1 min. The tubes were vortexed and centrifuged for 5 min at 16,000 × *g*, and the supernatant (cytoplasmic proteins) was transferred to a clean pre‐chilled tube on ice. The insoluble (pellet) fraction containing nuclei was suspended in ice‐cold NER. The tubes were then vortexed for 15 s. Samples were placed on ice for 40 min and vortexed for 15 s every 10 min. The tubes were centrifuged at 16,000 × *g* for 10 min. The supernatant (nuclear proteins) fraction was immediately transferred to a clean pre‐chilled tube on ice. The samples were analysed by immunoblotting.

### 2.6 Measurement of cellular ROS assay

LPS‐primed J774A.1 cells (0.1 × 10^6^ cells/well) were treated with or without TVE (10 μg/mL, 50 μg/mL, and 100 μg/mL) or NAC (3 mM) for 2 h and activated by ATP (5 mM) for 25 min. The medium was removed, and the cells were washed with 100 μL/well of 1× Buffer. Cells were incubated with DCFDA solution (20 μM) for 45 min at 37°C in the dark. The DCFDA solution was removed, and 100 μL/well of 1× supplemented buffer was added. The intracellular level of oxidative stress, with or without treatment, was measured by ROS production, according to the protocol of the cellular ROS assay kit (Abcam, ab113851). ROS levels were measured at Ex/Em = 485/535 nm using a BMG LabTech FLUOstar OPTIMA microplate reader.

### 2.7 Measurement ATPase activity of NLRP3

We generated purified mouse NLRP3 protein and used it for experiments. The purified mouse NLRP3 (BPS bioscience, 100189) (0.139 mg/mL) was incubated with TVE (50 μg/mL) or DMSO at 37°C for 30 min in the reaction buffer (100 mM Tris pH 7.8, 2.8 mM EDTA, 100 mM MgCl₂, 15 mM KCl, 655 mM NaCl). Ultrapure ATP (0.25 mM) was added, and the mixtures were further incubated for 40 min at 37°C. Hydrolysis of ATP (ATPase activity) by NLRP3 was determined by luminescent ADP detection performed by ADP‐Glo Kinase Assay (Promega) according to the manufacturer's instructions. The ATP activity was measured using a BMG LabTech FLUOstar OPTIMA microplate reader.

### 2.8 NF‐κB luciferase activity reporter gene assay

First, 293T cells (0.2 × 10^5^ cells/well) were seeded in a 96‐well white plate overnight. Subsequently, they were transfected with pGL4.32 Luciferase Reporter Vector (0.1 μg/well) using Lipofectamine 3000™ for 24 h. Then, nuclear factor‐kappa B (NF‐κB) activity was induced by tumour necrosis factor‐alpha (TNF‐α; 20 ng/mL) for 5 h with or without TVE (10 μg/mL, 50 μg/mL, and 100 μg/mL). Luciferase activity was measured using the Bright‐Glo™ Luciferase Assay System (Promega) according to the manufacturer's instructions. The luciferase activity was measured using a BMG LabTech FLUOstar OPTIMA microplate reader.

### 2.9 Immunoblot and Immunoprecipitation

Whole cell lysates were prepared using cold lysis buffer containing Tris–HCl pH 7.4 30 mM, EDTA 2 mM, NaCl 120 mM, KCl 2 mM, NP‐40 0.2%, glycerol 10%, and protease inhibitor cocktail. Cell lysates and supernatant proteins were separated using sodium dodecyl sulphate polyacrylamide gel electrophoresis and transferred to polyvinylidene difluoride membranes. The target proteins were visualized using enhanced chemiluminescence and detected by the Invitrogen iBright CL1500 imaging system. We conducted this analysis independently three times.

For immunoprecipitation, HEK 293FT cells (2 × 10^6^ cells) were transfected with NLRP3‐Myc. The cells were then lysed with lysis buffer and strongly vortexed for 20 s. The lysates were pre‐cleared and incubated with Myc and NEK7 antibodies overnight at 4°C. After incubating lysates with protein A/G beads for 1.5 h, the beads were rinsed and eluted. The samples and lysates were analysed by immunoblotting.

### 2.10 Asthma mouse model

All animal experimental protocols were approved by the Institutional Animal Care and Use Committee of Ajou University (IACUC2018‐0041). Six‐week‐old female BALB/c mice (Orient BIO, Seongnam, Korea) were maintained under specific‐pathogen‐free conditions. To induce neutrophilic asthma in wild‐type (WT) BALB/c mice, mice were sensitised with an intraperitoneal (i.p.) injection of 10 μg OVA (Ovalbumin, A5503, Sigma‐Aldrich) emulsified in aluminium hydroxide gel (InvivoGen, 77161) on days 0 and 7. From days 14–17, the mice were challenged with 6% OVA for 30 min using an ultrasonic nebulizer (n = 20) (NE‐SM1; Ktmed Inc., Seoul, South Korea). Mice were intranasally administered 10 μg of LPS (Sigma‐Aldrich, L2880) on days 15 and 17 in the neutrophilic asthma (NA) group (n = 20). To establish the effects of TVE and MCC950, the TVE group received 30 mg/kg of TVE orally, the MCC950 group received 50 mg/kg of MCC950 i.p., and the Dexamethasone (Dex) group received 1 mg/kg of Dex i.p. on days 14–17 before the challenge. For eosinophilic asthma (EA) in WT BALB/c mice, mice were sensitised with an i.p. injection of 10 μg OVA emulsified in aluminium hydroxide gel on days 0 and 7. On days 14–17, the mice were challenged with 6% OVA for 30 min using an ultrasonic nebulizer (n = 10). To establish the effects of TVE on EA, the TVE group received 30 mg/kg of TVE orally on days 14–17 before the challenge.

### 2.11 ELISA for bronchoalveolar lavage fluid (BALF)

We quantified and analysed human IL‐1β (DY201) in the supernatant collected from co‐cultured A549 cells and mouse IL‐1β (DY401), TNF‐α (DY410), and IL‐6 (DY406) from bronchoalveolar lavage fluid (BALF) obtained from mice using ELISA kit (R&D Systems), according to the manufacturer's instructions. The fluorescence intensity was measured at 450 nm using a BioTek Epoch 2 microplate spectrophotometer.

### 2.12 Haematoxylin and Eosin (H&E) staining and periodic acid‐Schiff (PAS) staining of lung tissue

A tissue sections from the right lower lung of mice were fixed on a slide with 4% paraformaldehyde. For H&E staining, after staining with haematoxylin for 3–5 min, counterstaining was performed by applying Eosin Y Solution for 2–3 min. For PAS staining, the tissues were stained with a periodic acid solution for 5 min and then stained with Schiff's solution for 15 min. The slide sections were observed for histopathological changes using a light microscope (Aperio CS2, Leica Biosystems, Wetzlar, Germany). We obtained images from tissue slides prepared using tissues from three or more mice, displayed representative images, and quantified the data by measuring total cell counts, immune cell counts and presented the results in quantified graphs.

### 2.13 Statistical analysis

The differences between treatment groups were assessed using one‐way analysis of variance (ANOVA) and Tukey's post hoc test, unless indicated otherwise. The differences between the treated and untreated cells in the in vitro assay were assessed using the Wilcoxon signed‐rank test. All statistical analyses were performed using SPSS software version 23.0 (SPSS Inc.), and a *p*‐value of <0.05 was considered significant.

## 3 RESULTS

### 3.1 TVE specifically suppresses NLRP3 inflammasome

**FIGURE 1:**
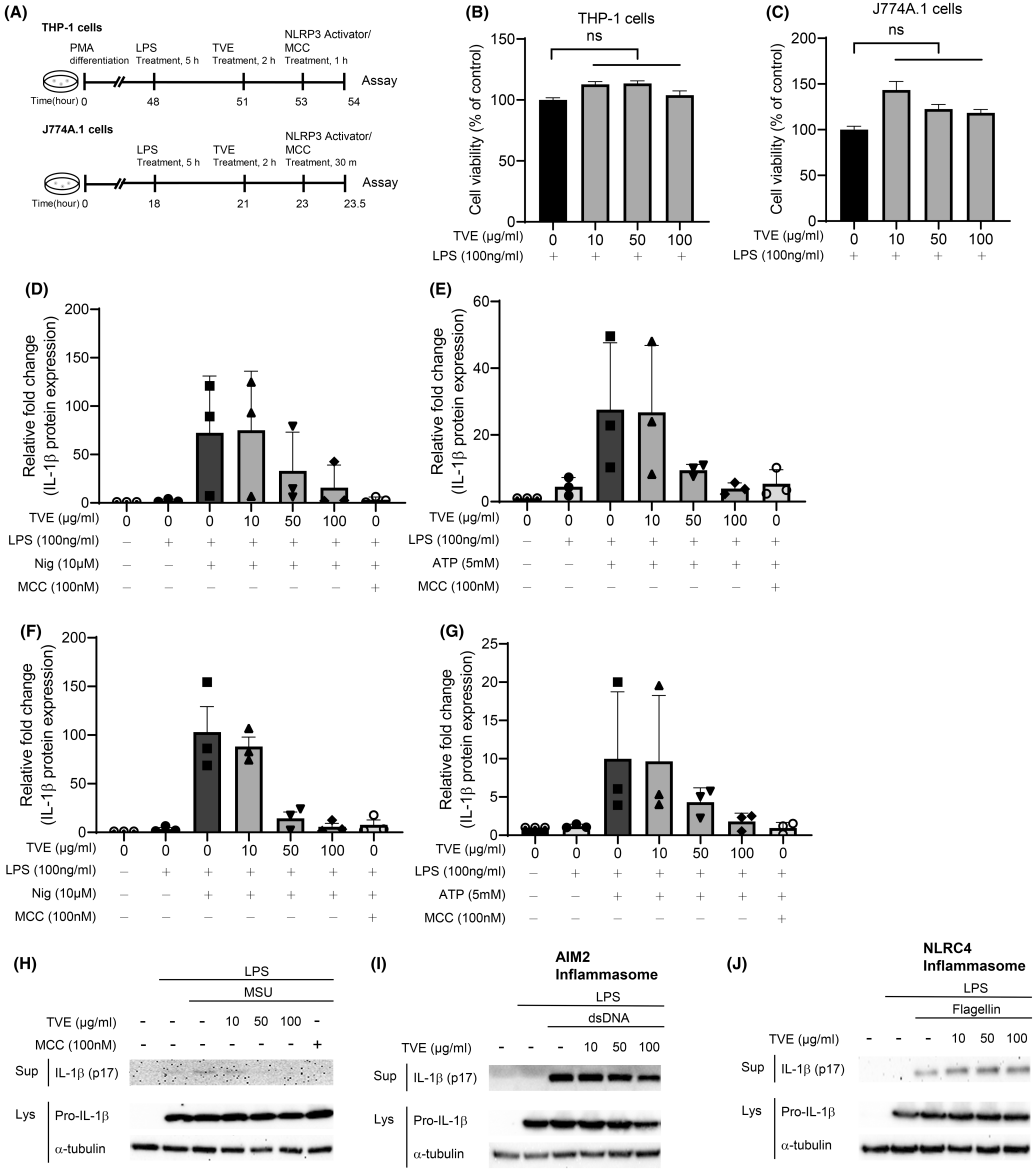
TVE specifically suppresses NLRP3 inflammasome. (A) Schematic outline of the NLRP3 inflammasome activation experiments in vitro. (B–C) PMA (500 nM)‐differentiated THP‐1 cells (B) and J774A.1 cells (C) were primed by LPS (100 ng/mL) for 3 h, before were treated with TVE for 2 h. Cell viability was analysed by EZ‐Cytox. (D–E) LPS‐primed THP‐1 cells were treated with TVE for 2 h and then stimulated for 1 h with nigericin (10 μM) (D) and ATP (5 mM) (E) with or without MCC950. (F–G) LPS‐primed J774A.1 cells were treated with TVE for 2 h and then stimulated for 30 min with nigericin (10 μM) (F), ATP (5 mM) (G) with or without MCC950. This data represents the investigation of the protein expression level of IL‐1β in supernatant, confirmed by immunoblot, using densitometry to assess intensity. (H‐J) IL‐1β (p17) and in the supernatants (Sup) and soluble lysates (Lys) were analysed by immunoblot. LPS‐primed J774A.1 cells were treated with TVE for 2 h and then stimulated for 3 h with MSU (100 μg/mL) (H) or dsDNA (2 μg/mL) (I) and flagellin (1.25 μg/mL) (J) by using Lipofectamine 3000™. One representative result of three independent experiments is shown. Values shown are reported as the means of technical triplicates ± SEM. One‐way ANOVA, Bonferroni post‐hoc test; *p < 0.05, **p < 0.01, ***p < 0.001, ****p < 0.0001. ATP, Adenosine triphosphate; IL‐1β, Interleukin‐1beta; LPS, Lipopolysaccharide; MCC, MCC950; Nig, Nigericin; n.s., not significant; TVE, *Trichospira verticillata* (L.) S.F. Blake Extract.

Figure 1A shows the experimental protocol for treatment with TVE and NLRP3 activators. LPS was administered for 3 h, followed by a 2 h pretreatment with TVE, and then treatment with NLRP3 inflammasome activators. First, to investigate whether TVE has cytotoxic effects, we examined the viability of THP‐1 and J774A.1 cells^15^ under the same conditions used in this experiment. Even at the maximum concentration of 100 μg/mL, TVE did not significantly affect cell viability (Figure 1B,C).^16^ To assess the effects of TVE on IL‐1β production, we treated LPS‐primed J774A.1 and THP‐1 cells with the NLRP3 inflammasome activators ATP, nigericin,^17^ and MSU (Figure 1H).^18^ The secretion of IL‐1β induced by NLRP3 activators considerably increased in the cell supernatant. However, this increase was attenuated by TVE treatment in a dose‐dependent manner, and this effect was comparable to that observed with MCC950 treatment,^19^ a well‐known NLRP3 inhibitor (Figure 1D–G, Figure S1A–D).^20^ The level of pro‐IL‐1β in the lysates remained unchanged (Figure S1A,B). Similar results were obtained in THP‐1 cells (Figure S1C,D). We also investigated the impact of TVE treatment on the activation of other inflammasomes, such as AIM2 and NLRC4. As shown in Figure 1I,J, TVE treatment had no effect on the production of IL‐1β induced via AIM2 and NLRC4 inflammasome activation by double‐stranded DNA and flagellin, respectively. These results suggest that TVE specifically suppressed the NLRP3 inflammasome.

### 3.2 TVE does not affect NF‐κB signalling

**FIGURE 2:**
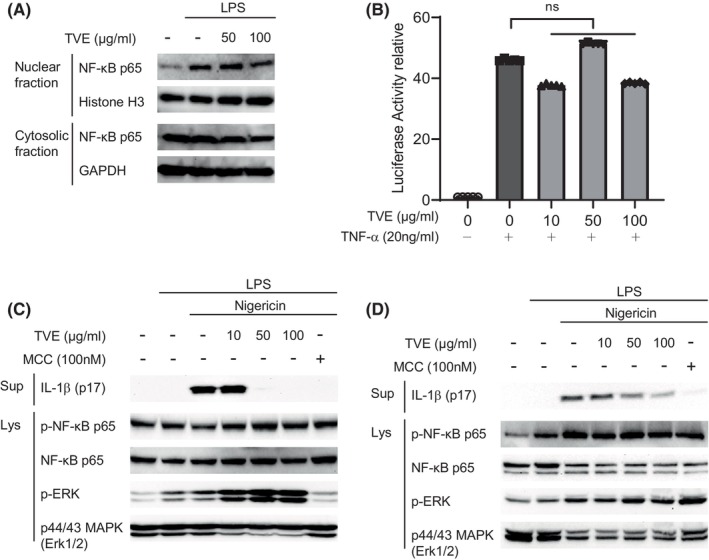
TVE does not affect the NF‐κB signalling pathway. (A) Nuclear fractionation indicated translocation of NF‐κB p65. LPS‐primed J774A.1 cells were treated with TVE for 2 h. The nuclear and cytosolic fractions in soluble lysates were analysed by immunoblot. (B) The pGL4.32 Luciferase Reporter vector‐transfected 293 T cells were treated simultaneously with TNF‐α (20 ng/mL) and TVE for 5 h. Luciferase activity of NF‐κB was measured using the Promega Bright‐Glo Luciferase assay system. (C–D) LPS‐primed J774A.1 cells (C) and LPS‐primed THP‐1 cells (D) were treated with TVE for 2 h and then stimulated for 30 min with nigericin (10 μM). The supernatants (Sup) and soluble lysates (Lys) were analysed by immunoblot. One representative result of three independent experiments is shown. Values are shown reported as the means of technical triplicates ± SEM. One‐way ANOVA, Bonferroni post‐hoc test; *p < 0.05, **p < 0.01, ***p < 0.001, ****p < 0.0001. LPS, Lipopolysaccharide; n.s., not significant. TVE, *Trichospira verticillata* (L.) S.F. Blake Extract.

We then investigated the molecular mechanism underlying the suppressive activity of TVE on the NLRP3 inflammasome. The production process of IL‐1β is divided into two steps. The first step is the priming phase, where the activation of NF‐κB leads to an increase in the transcription of pro‐IL‐1β.^21^, ^22^ The second step is the activation phase, where an active substance induces inflammasome formation. To investigate whether TVE treatment suppressed the NLRP3 inflammasome by reducing NF‐κB activity during the priming step, we analysed the nuclear translocation of the p65, one of the NF‐κB subunits. Treatment with LPS increased the nuclear translocation of p65 through toll‐like receptor 4 (TLR4), whereas treatment with TVE had little effect on the translocation of p65 (Figure 2A). To confirm that TVE has no direct effect on NF‐κB activity, we performed an NF‐κB luciferase reporter gene assay and found that TVE did not affect TNF‐α‐induced NF‐κB signalling (Figure 2B). Furthermore, we confirmed that treatment with TVE did not affect the level of phospho p65, an indicator of NF‐κB activity, even after treatment with NLRP3 activators (LPS and nigericin). We also confirmed that the level of p‐ERK, the downstream target of NF‐κB, was also unchanged in both J774A.1 and THP‐1 cells treated with TVE (Figure 2C,D). These findings indicate that treatment with TVE suppressed the NLRP3 inflammasome without affecting NF‐κB signalling.

### 3.3 TVE prevents the binding of NEK7 to NLRP3

**FIGURE 3:**
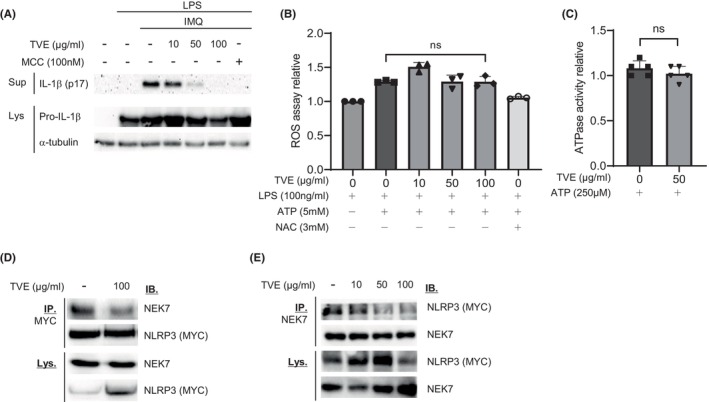
TVE suppresses NLRP3 inflammasome activation regardless of K^+^ efflux, ROS and ATPase activity. (A) LPS‐primed J774A.1 cells were treated with TVE for 2 h and then stimulated with IMQ (200 μM) for 1 h. (B) LPS‐primed J774A.1 cells were treated with or without TVE or NAC for 2 h and then stimulated with ATP (5 mM) for 25 min. ROS levels are detected by a microplate reader using a DCFDA solution (20 μM). (C) The amount of NLRP3‐mediated ATP converted into ADP with TVE was determined by luminescence using the ADP‐Glo Assay. (D) and (E) The NLRP3‐Myc transfected‐HEK 293FT cells were treated with or without TVE for 2 h. The NLRP3‐NEK7 interaction was analysed by immunoprecipitation and immunoblot. One representative result of three independent experiments is shown. Values are shown reported as the means of technical triplicates ± SEM. One‐way ANOVA, Bonferroni post‐hoc test; *p < 0.05, **p < 0.01, ***p < 0.001, ****p < 0.0001. LPS, Lipopolysaccharide; n.s., not significant; NAC, N‐acetyl‐l‐cysteine; ROS, reactive oxygen species; TVE, *Trichospira verticillata* (L.) S.F. Blake Extract.

Potassium efflux and increased intracellular ROS levels play essential roles in NLRP3 inflammasome activation. Therefore, NLRP3 inflammasome activity can be suppressed by reducing intracellular ROS^23^, ^24^ or preventing potassium efflux.^25^ To investigate whether TVE could suppress NLRP3 by reducing intracellular ROS or preventing potassium efflux, we first treated LPS‐primed J774A.1 cells with imiquimod,^26^ which activates the NLRP3 inflammasome regardless of potassium efflux, followed by treatment with TVE. As shown in Figure 3A, TVE treatment inhibited NLRP3 activation by imiquimod in a concentration‐dependent manner, indicating that the suppressive effect of TVE on the NLRP3 inflammasome is not attributed to potassium efflux. To determine whether TVE inhibited NLRP3 by reducing intracellular ROS levels, we measured ROS levels after treatment with TVE. While intracellular ROS levels increased upon treatment with ATP, they remained unchanged after treatment with TVE and were reduced by treatment with NAC, a known ROS scavenger, to levels similar to those before treatment with ATP (Figure 3B). Many newly developed NLRP3 inhibitors bind to the Nacht domain of NLRP3 to inhibit its ATPase activity.^27^, ^28^ Therefore, we performed an in vitro ATPase assay to investigate whether TVE inhibits ATPase activity and found that TVE did not affect ATPase activity (Figure 3C).

The NEK7‐NLRP3 interaction is essential for the NLRP3 inflammasome assembly.^29^ To investigate whether TVE affected this interaction, we performed an immunoprecipitation experiment. As shown in Figure 3D,E, upon TVE treatment, the binding between the two proteins substantially decreased, indicating that TVE inhibited the binding of NLRP3 to NEK7,^30^ thereby suppressing the activation of NLRP3 inflammasome.

### 3.4 TVE blocks NLRP3 inflammasome assembly by hindering ASC oligomerization

**FIGURE 4:**
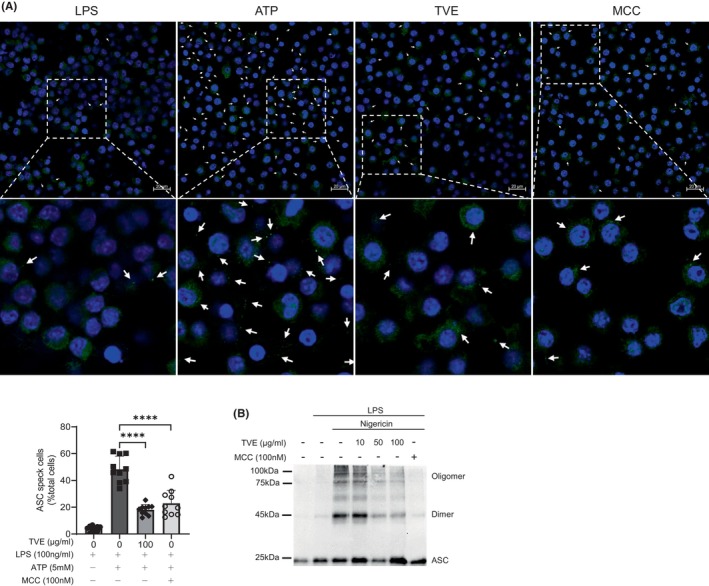
TVE blocks NLRP3 inflammasome assembly by hindering ASC oligomerization. (A) One representative ASC speck (indicated by arrow) images of at least 10 images are shown. LPS‐primed J774A.1 cells were treated with TVE for 2 h and then stimulated with ATP (5 mM) for 30 min. (B) LPS‐primed THP‐1 cells were treated with TVE for 2 h and then stimulated with nigericin (10 μM) for 30 min. ASC oligomerization in the cross‐linked cytosolic pellet was analysed by immunoblot. Scale bar, 20 μm. One representative result of three independent experiments is shown. Values are shown reported as the means of 10 replicates ± SEM. One‐way ANOVA, Bonferroni post‐hoc test; *p < 0.05, **p < 0.01, ***p < 0.001, ****p < 0.0001. ASC, apoptosis‐associated speck‐like protein; LPS, Lipopolysaccharide; n.s., not significant; TVE, *Trichospira verticillata* (L.) S.F. Blake Extract.

During NLRP3 activation, ASC, an inflammasome component, translocates to the detergent‐insoluble fraction, and ASC specks are formed due to the assembly of ASC oligomers.^31^, ^32^ Therefore, we investigated the effects of TVE on ASC speck formation in J774A.1 cells.^33^ MCC950 was used as a positive control to compare the degree of ASC oligomerization. ATP‐treated J774A.1 cells exhibited a notable increase in ASC speck formation, and upon treatment with TVE or MCC950, there was a remarkable reduction in the number of ASC specks (Figure 4A). To confirm these results, we measured ASC oligomerization, another hallmark of inflammasome activation in THP‐1 cells. Similar to the ASC speck results, ASC oligomerization substantially decreased after treatment with TVE or MCC950 (Figure 4B),^34^ suggesting that TVE inhibits NLRP3 inflammasome activation by suppressing ASC oligomerization.

### 3.5 TVE also suppresses NLRP3 inflammasome in lung epithelial cells

**FIGURE 5:**
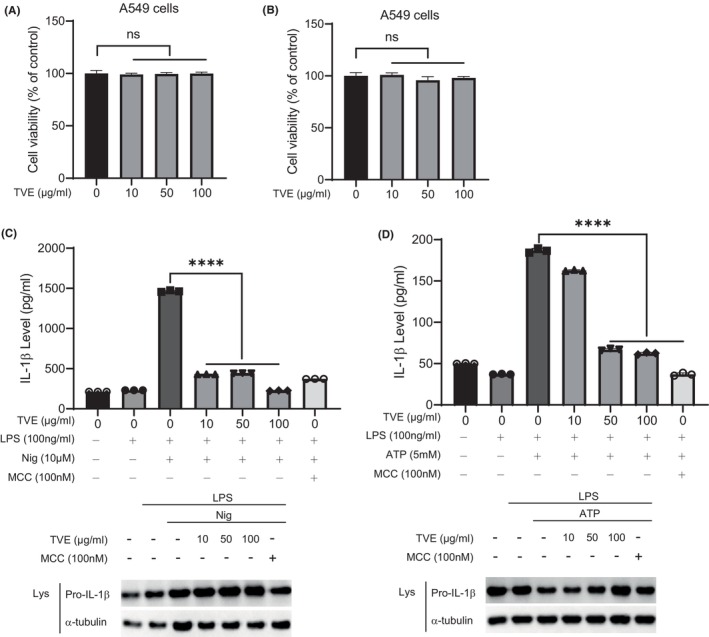
TVE inhibits NLRP3 inflammasome in lung epithelial cells. (A–B) A549 cells were treated with TVE for 2 h (A), or overnight (B). Cell viability was analysed by EZ‐CYTOX. (C–D) A549 cells and PMA (500 nM)‐differentiated THP‐1 cells were co‐cultured and primed by LPS (100 ng/mL) for 3 h, before they were treated with TVE for 2 h and stimulated for 30 min with nigericin (10 μM) (C), and ATP (5 mM) (D). The level of IL‐1β was evaluated by ELISA and the lysate was analysed by immunoblotting. One representative result of three independent experiments is shown. Values are shown reported as the means of technical triplicates ± SEM. One‐way ANOVA, Bonferroni post‐hoc test; *p < 0.05, **p < 0.01, ***p < 0.001, ****p < 0.0001, n.s. not significant.

We sought to investigate whether the inhibitory effects of TVE on the NLRP3 inflammasome mechanisms could be applicable in neutrophilic asthma (NA), which is associated with the NLRP3 inflammasome. Given that the airway epithelium is the first site for initiating the immune response during severe asthma, we initiated experiments using the lung epithelial cell line, A549. Firstly, to assess toxicity of TVE on A549 cells, we treated A549 cells with TVE for 2 h or overnight. Similar to Figure 1B,C, TVE treatment did not affect the viability of A549 cells (Figure 5A,B). However, under our experimental conditions, NLRP3‐dependent IL‐1β secretion was not observed in A549 cells. A recent paper reported that IL‐1β is secreted from A549 cells when co‐cultured with immune cells.^35^ Moreover, as co‐culture systems are considered more representative of actual asthma conditions, we introduced a co‐culture system in our experiments. We co‐cultured A549 cells with PMA‐differentiated THP‐1 cells, administered LPS for 5 h, and treated them with TVE for 2 h, followed by the treatment of NLRP3 inflammasome activators. NLRP3 activator treatment did not increase IL‐1β secretion in A549‐only cells; however, it slightly increased IL‐1β secretion in THP‐1 cells at the same number as those used in the co‐culture (Figure S2). Interestingly, in the co‐culture of A549 and THP‐1 cells, a significantly higher amount of IL‐1β was secreted compared to the A549‐alone or THP‐1‐alone groups, suggesting that A549 cells can be activated during co‐culture with THP‐1, leading to the production of a substantial amount of IL‐1β. As shown in Figure 5C,D, TVE effectively suppressed NLRP3‐dependent IL‐1β secretion in the co‐culture system. Taken together, TVE can block NLRP3 not only in immune cells but also in epithelial cells without toxicity.

### 3.6 TVE treatment significantly alleviates asthma symptoms in an NA mouse model

**FIGURE 6:**
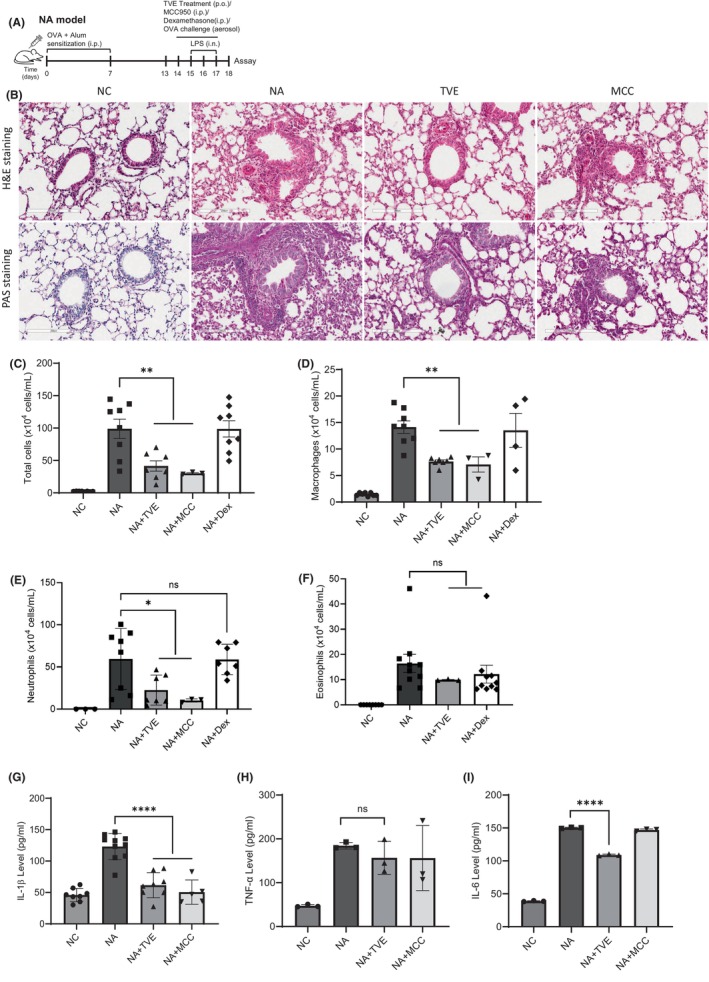
TVE treatment significantly alleviates asthma symptoms in a neutrophilic asthma mouse model. (A) Schematic outline of the neutrophilic asthma mouse model. (B) Lung tissues were stained with haematoxylin and eosin (H&E) (upper panel) or PAS (lower panel). Scale bar, 200 μm. One representative image of three to five slides is shown. (C–F) The numbers of total cells, macrophages, neutrophils and eosinophils in the bronchoalveolar lavage fluid (BALF) were measured. (G–I) The levels of IL‐1β, TNF‐α and IL‐6 were evaluated by ELISA in the BALF (*n* = 5). All data are presented as the mean ± SEM. One‐way ANOVA, Bonferroni post‐hoc test; *p < 0.05, **p < 0.01, ***p < 0.001, ****p < 0.0001. Dex, Dexamethasone; n.s., not significant; NA, neutrophilic asthma; NC, Control; TNF‐α, Tumour necrosis factor‐alpha; IL‐6, Interleukin‐6.

To determine whether TVE treatment inhibits the NLRP3 inflammasome in vivo, we used asthma mouse models. Figure 6A shows the sensitization and challenge protocols for generating the eosinophilic asthma (EA) and neutrophilic asthma (NA) mouse model.^36^, ^37^ We performed H&E staining and PAS staining to identify the pathological changes in lung inflammation and mucus production after treatment with TVE in NA mouse model. As shown in Figure 6B, an accumulation of infiltrated cells was observed around the airways in the lung tissue of the NA mouse model,^38^ whereas the number of infiltrating cells was significantly reduced in the lung tissue upon treatment with TVE or MCC950. Similarly, the analysis of the mucus produced by airway epithelial cells in the PAS staining experiment revealed a significant increase in mucus in the NA model; however, treatment with TVE or MCC950 strongly inhibited mucus production (Figure 6B). Moreover, the overall cell count and number of macrophages and neutrophils in the BALF from the NA model significantly increased owing to the increase in infiltrating cells, which was not suppressed by Dex, indicating steroid resistance; however, these increased immune cell numbers were significantly reduced by treatment with TVE or MCC950 (Figure 6C–F). If TVE suppresses NLRP3 and plays a role in the NA mouse model, which is associated with NLRP3 inflammasome, it should have no effect in the EA model, which is unrelated to NLRP3 inflammasome. Therefore, we treated the EA mouse model with TVE (Figure S3A) and measured the infiltrated immune cells count. Unlike the NA model, treating the EA model with TVE did not make any difference in the counts of macrophages, neutrophils, and eosinophils (Figure S3B–E), implying that TVE functions exclusively in NA by inhibiting the NLRP3 inflammasome. The proinflammatory cytokines, IL‐1β, IL‐6 and TNF‐α, were also appreciably increased in the NA model. Although treatment with TVE notably reduced the level of IL‐1β, it had no substantial impact on the secretion of IL‐6 and TNF‐α. Similarly, in the group treated with MCC950, only the IL‐1β was significantly reduced; the other cytokines were not considerably affected (Figure 6G–I). Similar to the infiltrated immune cells data, in the EA model, there was no change in the secretion of pro‐cytokines even after treating with TVE (Figure S3F).

## 4 DISCUSSION

The increase in IL‐1β has been reported as a key factor in steroid‐resistant neutrophilic asthma, and the elevation of IL‐1β is associated with the development of steroid resistance.^7^, ^39^ NA patients exhibit significantly higher level of IL‐1β‐related proteins and gene expressions compared to EA patients, and this increase in IL‐1β is positively correlated with neutrophil counts and IL‐8 level.^29^ Additionally, in the NA mouse model, steroids such as Dex are ineffective, but IL‐1β blockers like anakinra (IL‐1 receptor antagonist) have been reported to reduce airway neutrophilic inflammation.^34^, ^37^ Therefore, the development of therapeutic interventions for NA utilising the inhibition mechanism of IL‐1β is a highly intriguing topic. While IL‐1β can be secreted through various inflammasomes activated by various triggers, the most extensively researched and closely associated with asthma is the NLRP3 inflammasome. Its abnormal or chronic activation is directly associated with the excessive secretion of IL‐1β and numerous inflammatory diseases. Consequently, targeting NLRP3 inflammasome when aiming at IL‐1β as a therapeutic target for severe asthma has proven to be effective.

The activation model of the NLRP3 inflammasome is divided into two stages: the priming step, where the transcription of inflammatory proteins increases through NF‐κB, and the activation step, where the inflammasome complex is formed by triggers such as K+ efflux and ROS elevation.^36^ Therefore, blocking NF‐κB signalling or removing the triggers of the activation step can be a method to inhibit NLRP3. However, these approaches are predicted to have significant side effects due to their essential roles in various intracellular responses. Consequently, research on small molecules that specifically reduce NLRP3 has been actively pursued, and some small molecules have been reported to exhibit dramatic and selective effects on NLRP3 suppression. However, none of them has currently received approval from the Food and Drug Administration (FDA) or other regulatory.^40^ In this way, although chemical and bio‐based drug development is the mainstream approach, it is plagued by intense competition and severe cytotoxicity issues, thus prompting the development of natural product‐based drugs as alternatives.

In particular, investigating the NLRP3 inhibitory properties of natural resources that have never been researched before is beneficial for the identification of new substances and materials that are not covered by existing patents, which makes it a potential field. Therefore, we analysed the anti‐inflammatory properties of over 200 natural products derived from Costa Rica and Nicaragua and identified several candidates with strong anti‐inflammatory responses and no cytotoxicity (data not shown). Among them, *Trichospira* was selected as a key anti‐inflammatory target because of its outstanding anti‐inflammatory activity and lack of toxicity. A previous study reported that lactone compounds extracted from *Trichospira* have antiplasmodial activity.^1^

In this study, after screening for natural products that could inhibit the activity of the NLRP3 inflammasome, which generates active IL‐1β, an important mediator of inflammatory responses, we analysed the underlying molecular mechanism of the inhibitory effect by the methanol extract of *Trichospira verticillata*. First, we confirmed that TVE was not toxic in mouse and human cell lines under our experimental conditions. Second, we observed a notable reduction in the amount of IL‐1β secretion induced by NLRP3 inflammasome when treated with TVE. Moreover, we compared the inhibitory effects of TVE with those of MCC950, an NLRP3 inhibitor, and observed similar effects at high concentrations. Finally, we found that treatment of TVE completely suppressed the binding of NLRP3 to NEK7, which is essential for inflammasome assembly.

Since NLRP3 inflammasome has been reported to be highly activated in neutrophilic asthma (NA), we investigated whether TVE's NLRP3 inhibitory function could play a role in NA. As the interaction between epithelial cells and immune cells is important in asthma, we used A549 cells to investigate the secretion of IL‐1β after TVE treatment in initial experiments, but we were unable to detect any IL‐1β secretion in our conditions. Upon reviewing recent literature, we found a paper reporting IL‐1β secretion was observed in in vitro experiments, in which inflammatory cells are simulated to infiltrate lung epithelial cells similar to in vivo conditions.^35^ Subsequently, in our co‐culture experiments, TVE significantly reduced an NLRP3‐dependent IL‐1β secretion in A549 cells co‐cultured with THP‐1 cells. After confirming that TVE functions effectively in epithelial cells, we finally conducted experiments using NA model to investigate TVE's function in vivo. TVE treatment significantly reduced the infiltrated immune cells and IL‐1β secretion observed in NA, and this reduction was comparable to the effect of MCC950. Interestingly, in the NA mouse model, levels of TNF‐α and IL‐6 were also increased, but TVE treatment did not significantly reduce TNF‐α. Based on these results, it can be predicted that the increase in IL‐1β due to the activation of NLRP3 in NA may be a downstream signal of TNF‐α. Therefore, it is speculated that treatment with TNF‐α inhibitors may effectively reduce the level of IL‐1β in NA.

There are some limitations in our study. Firstly, this experiment did not confirm whether TVE directly binds to NLRP3 to block its activity. Therefore, further studies are required to explore the constituents and components of TVE that bind directly to NLRP3 and inhibit its activity. Secondly, our study primarily focused on identifying new substances that inhibit the secretion of IL‐1β through NLRP3 in severe asthma. However, severe asthma is a complex disease influenced by various genetic and environmental factors. Considering the interactions between IL‐1β and other cytokines would provide a more effective treatments for severe asthma. Despite these limitations, our research indicates the potential of TVE as a therapeutic agent in NA. In addition, *Trichospira* belongs to the family *Asteraceae*, which is used as a medicinal plant in Asian countries. Other *Asteraceae* plants, such as *Tussilago farfara L*. and *Aster tataricus*, are already used to treat asthma and inflammation. As *Tanacetum parthenium* can be used to alleviate inflammation, additional analysis of *Trichospira* to explore its potential as a medicinal plant owing to its anti‐inflammatory properties and its efficacy as a therapeutic agent for asthma is considerably valuable.

## AUTHOR CONTRIBUTIONS

**Hyeyun Yang:** Formal analysis (lead); investigation (lead); methodology (lead); writing – original draft (equal); writing – review and editing (equal). **Gunwoo Park:** Investigation (equal); methodology (equal). **Sojung Lee:** Investigation (equal); methodology (equal). **Sumin Lee:** Investigation (equal); methodology (equal). **YeJi Kim:** Investigation (equal); methodology (equal). **Nelson A. Zamora:** Resources (equal). **Dong‐Keun Yi:** Methodology (equal); resources (equal). **Soo‐Yong Kim:** Investigation (equal); resources (equal). **Chun Whan Choi:** Formal analysis (equal); investigation (equal); methodology (equal). **Sangho Choi:** Methodology (equal); resources (equal). **Yong Hwan Park:** Funding acquisition (lead); project administration (lead); supervision (lead); writing – original draft (supporting).

## FUNDING INFORMATION

This work was supported by the National Research Foundation of Korea (NRF) grant funded by the Korean government (MSIT) (NRF‐2022R1F1A106940312 and RS‐2023‐00217595). This research was carried out with support from the International Center for Biological Materials Research (IBMRC) by signing an MOU.

## CONFLICT OF INTEREST STATEMENT

The authors declare no conflict of interest.

## Supporting information


Figure S1.

Figure S2.

Figure S3.

## Data Availability

The data supporting the findings of the present study are available from the corresponding author upon reasonable request. Some data may not be made available because of privacy or ethical restrictions.
