# Supplementary figures and images for: Anti‐inflammatory effect of Trichospira verticillata via suppression of the NLRP3 inflammasome in neutrophilic asthma
Source: J Cell Mol Med. 2024 Apr 26;28(8):e18356. doi: 10.1111/jcmm.18356 (PMC11048967; doi:10.1111/jcmm.18356)

Supplementary Figure 1

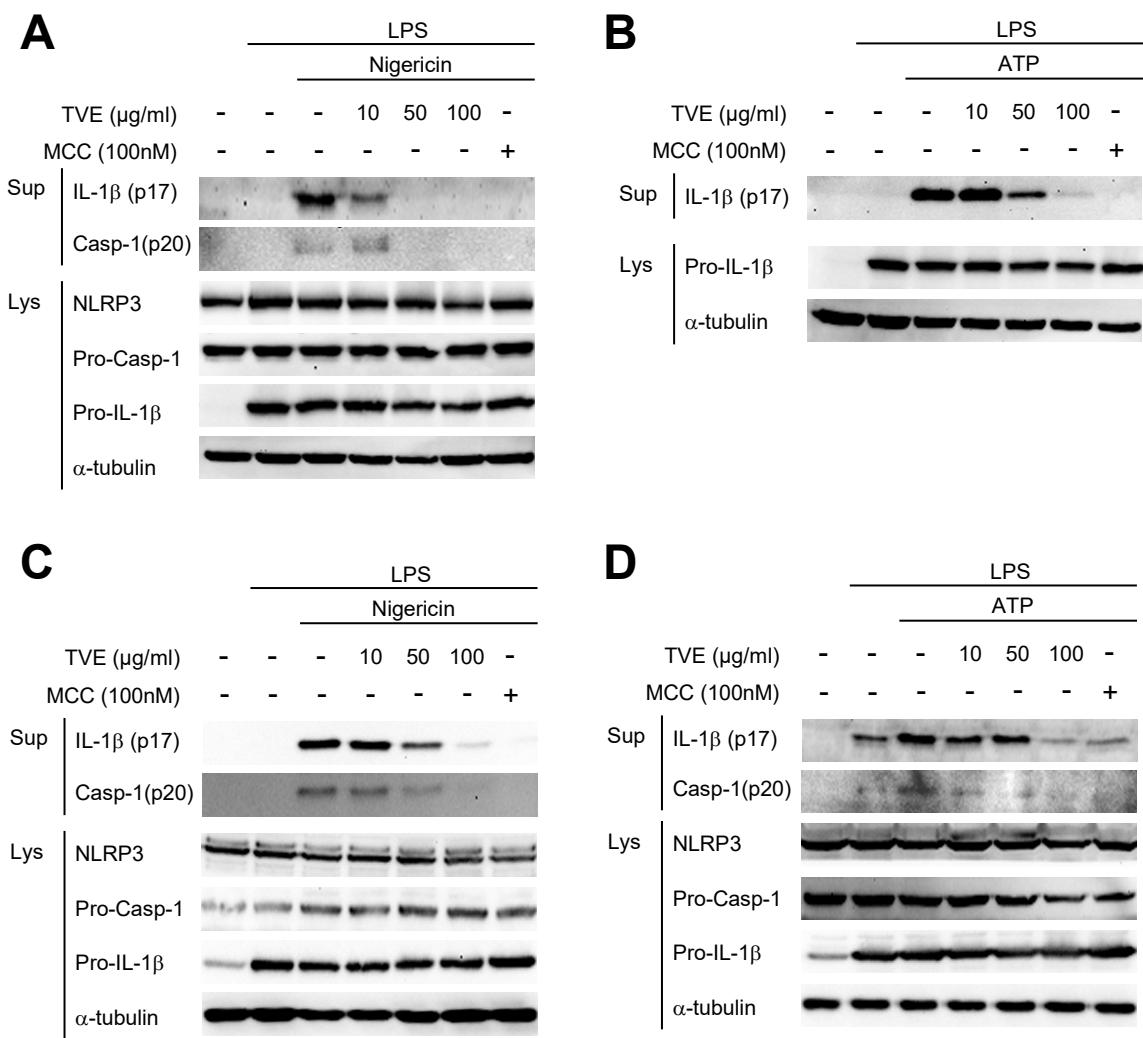

Supplement: Supplementary file 1 — Figure S1. [file JCMM-28-e18356-s003.pdf]

Supplementary Figure 2

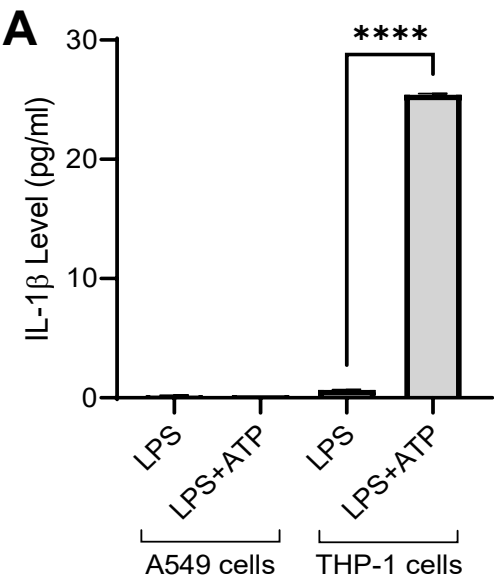

Supplement: Supplementary file 2 — Figure S2. [file JCMM-28-e18356-s001.pdf]

Supplementary Figure 3

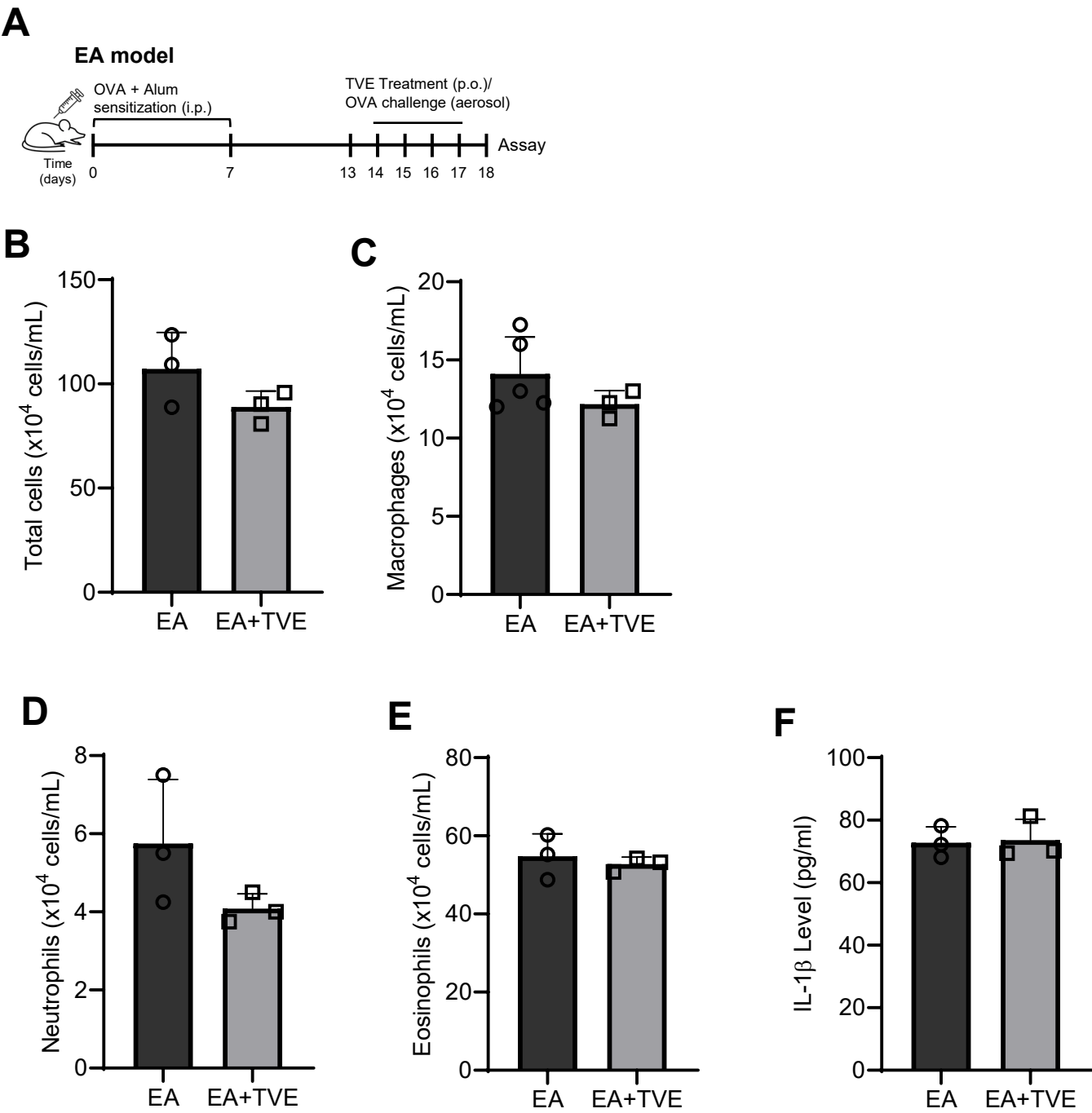

Supplement: Supplementary file 3 — Figure S3. [file JCMM-28-e18356-s002.pdf]
